# Genetic Diversity and Population Genetic Structure of Endemic Schizothoracinae Fishes in the Upper Yellow River and Its Adjacent Waters

**DOI:** 10.1002/ece3.72813

**Published:** 2025-12-18

**Authors:** Xiu Feng, Xiaoling Wang, Ren Zhu, Yintao Jia, Xiaoyun Sui, Yu Zhuo, Junle Li, Yifeng Chen

**Affiliations:** ^1^ Institute of Hydrobiology Chinese Academy of Sciences Wuhan China; ^2^ University of Chinese Academy of Sciences Beijing China; ^3^ State Key Laboratory of Breeding Biotechnology and Sustainable Aquaculture, Institute of Hydrobiology Chinese Academy of Sciences Wuhan China

**Keywords:** genetic diversity, mitochondrial DNA, population genetic structure, Schizothoracinae fishes, the upper Yellow River

## Abstract

The endemic fishes in the upper Yellow River and its adjacent waters on the Qinghai‐Tibet Plateau are very sensitive and vulnerable to human activities and climate changes. However, the status of populations, such as genetic diversity and population genetic structure, remains unclear, limiting further conservation and utilization of their natural resources. Here, genetic diversity and population genetic structure of the highly specialized Schizothoracinae fishes were analyzed by using concatenated mitochondrial COI and D‐loop sequences of 517 samples from 21 geographic populations in Qinghai Lake (QHL), the canyon section and the river source section of the upper Yellow River (CUYR and RUYR), Qaidam River (DQDR), and Golmud River (GMR). The results showed that populations from CUYR, RUYR, and QHL showed higher genetic diversity than the DQDR populations, and the GMR population exhibited the lowest haplotype diversity but high nucleotide diversity. In the upper Yellow River, dam‐affected populations with low resource supplementation showed reduced genetic diversity, while those with frequent stocking events exhibited comparable genetic diversity relative to undisturbed populations. All samples were divided into three distinct phylogenetic lineages, with QHL and DQDR samples belonging to only one single and distinct lineage each, and GMR samples included in both lineages, with CUYR and RUYR samples assigned to all lineages. Low genetic differentiation was detected between GMR and RUYR, QHL and CUYR, and CUYR and RUYR. These findings indicated that the population genetic structure of endemic Schizothoracinae fishes in the upper Yellow River and its adjacent waters was primarily shaped by hydrological connectivity and isolation caused by historical geological events, and the genetic diversity was influenced by dam construction and stocking activities.

## Graphical abstract

The upper Yellow River and Qinghai Lake populations exhibited higher genetic diversity than the isolated populations from the Qaidam River and Golmud River, with all three lineages present only in the upper Yellow River versus one or two lineages in others. Our results indicated that habitat isolation and connectivity caused by paleogeological events primarily drove the formation of the population genetic structure, and human activities such as dam construction and stocking activities have had a profound impact on population genetic diversity.

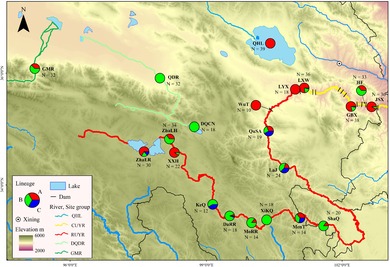

## 1 Introduction

Genetic diversity refers to the total genetic variations within the gene pool of a species, including diversity in alleles, genotypes, and differences among individuals, and serves as the foundation for a species' ability to adapt to environmental changes, resist diseases, and maintain long‐term survival and evolution. The population genetic structure refers to the distribution and composition of genetic variation within and between populations of a species, including the frequencies of alleles and genotypes and their differences among populations or different individuals within a population, which may be influenced by gene flow, genetic drift, natural selection, geographic isolation, and reproductive barriers. Studying population genetic diversity and genetic structure can provide a theoretical basis for the conservation, utilization, and management of genetic resources (Salgotra and Chauhan 2023). For example, it can help identify populations that are at risk of extinction due to low genetic diversity or limited gene flow, thus guiding conservation efforts, such as the establishment of protected areas or the translocation of individuals to enhance genetic diversity (Frankham 2010). It can also help in identifying genetically distinct populations that may harbor unique traits that may be selected and combined to create new varieties with improved performance (Glaszmann et al. 2010). Furthermore, the results can enable an understanding of the origins, migration patterns, and adaptive changes of a species (Feng, Liu, et al. 2023).

The unique geographical, climatic, and environmental characteristics of the Qinghai‐Tibet Plateau (QTP) have contributed to the development of a distinct, diverse, and fragile plateau water ecosystem and aquatic biodiversity. The fish fauna on the QTP is mainly composed of the subfamily Schizothoracinae and the genus *Triplophysa* (Wu and Tan 1991). The Schizothoracinae fishes are divided into three evolutionary grades including the highly specialized grade (characterized by no barbels and scales, one or two rows of pharyngeal teeth), the specialized grade (one pair of barbels, two rows of pharyngeal teeth, moderately degenerated scales), and the primitive grade (two pairs of barbels, three or four rows of pharyngeal teeth, less degeneration of scales), representing three special stages during the evolutionary course of the plateau (Cao et al. 1981). Most of the Schizothoracinae fishes in the upper Yellow River and surrounding waters (i.e., *Gymnocypris eckloni* , *G. przewalskii* , *Schizopygopsis pylzovi* , *S. kessleri* , *Chuanchia labiosa* , *Platypharodon extremus* ) are endemic and belong to the highly specialized grade. However, *Gymnodiptychus pachycheilus* belongs to the specialized grade. They have been listed as Endangered (EN) or Vulnerable (VU) species by the Red List of China's vertebrates (Jiang et al. 2016), due to the decline of natural populations (Jia et al. 2019). Some of them hold considerable commercial significance and serve as a critical protein source for local people. Hence, the conservation of these fishes is an urgent task that has attracted considerable attention. Although a few studies have investigated their phylogenetics (He et al. 2004; Zhao et al. 2005; He and Chen 2007; Rozimov et al. 2025), critical population genetic information, such as genetic diversity and genetic structure, remains unclear, limiting the further conservation and utilization of their natural populations.

Paleoclimate and paleogeological events are the primary factors shaping the genetic diversity and genetic structure of extant species (Yan et al. 2013). Since the Middle Pleistocene, the formation of the upper Yellow River and its separation from several adjacent endorheic basins have been one of the most significant geological events on the northeast QTP (Li et al. 2000; Qi 2016). Driven by tectonic uplift and river headward erosion, the upper Yellow River cut through the Jishi canyon and arrived in Hualong‐Guide (about 1.1 million years ago), then cut through the Longyang canyon and appeared in the Gonghe basin (about 0.15 million years ago), and finally cut through Nanshan, Guinan, and western Qinling and reached the source of the Yellow River passing Ruoergai basin (about 0.03 million years ago) (Zhang et al. 2003). The formation of the upper Yellow River was accompanied by its disconnection from several basins, such as Qinghai Lake and Gonghe Basin. These paleogeological events may have been the primary drivers in shaping the genetic diversity and genetic structure of fish species in this region, but the relationship between them remains poorly understood.

Human activities in the upper Yellow River and its adjacent water bodies, such as the construction of water resources and hydropower engineering in the canyon section of the upper Yellow River and the Golmud River, overfishing in Qinghai Lake, and human‐induced biological invasions, have led to a sharp decline in fish resources in this region (Jia et al. 2019, 2020, 2024). Some fish species, such as *C. labiosa* and *P. extremus* , which are originally distributed in the section from Liujiaxia to Longyangxia, have retreated to the section above Longyangxia (Zhao et al. 2020). The theoretical consequence of these impacts would be a decline in population genetic diversity and genetic differentiation among populations in fragmented habitats. However, since the 2000s, consecutive stock enhancement activities have supplemented fish resources in this region. For example, a total of 220 million individuals of *G. przewalskii* were released into Qinghai Lake from 2002 to 2023 (Fu et al. 2024). Approximately 15.81 million individuals of *G. eckloni* whose parents originated from the source section of the Yellow River were primarily released into the canyon sections of the upper Yellow River from 2009 to 2020, with varying stocking frequencies across different reservoirs (Li et al. 2021). However, the effects of anthropogenic activities on the genetic diversity and population structure of fishes in this region remain unclear.

Mitochondrial DNA (mtDNA), due to its maternal inheritance, rapid evolution, and high copy number, has been widely used as a molecular marker for genetic diversity and structure analysis (Galtier et al. 2009). A fragment of approximately 650 bp of the mitochondrial cytochrome oxidase subunit I (COI) gene has been proposed as a rapid and reliable barcoding marker for species identification across the entire animal kingdom (Hebert et al. 2003) and population genetic analysis with close genetic relationships (Feng et al. 2018). In comparison, the mtDNA control region (D‐loop) has a faster mutation rate and more accumulated mutations and has also been widely used for studying population genetic diversity and structure among different populations of the same species (Feng, Zhu, et al. 2023). The D‐loop is commonly used in combination with other mitochondrial genes for fish population genetic analysis (Zhang et al. 2020).

In this study, a total of 517 samples of endemic Schizothoracinae fishes were collected from 21 sites of the upper Yellow River and its adjacent waters on the northeast QTP, and their mitochondrial COI and D‐loop sequences were analyzed. This study aimed to: (i) uncover the genetic relationships among different Schizothoracinae fishes; (ii) investigate the genetic diversity and genetic structure and the degree of genetic differentiation within and among geographical populations; (iii) discuss the factors driving the genetic diversity and structure.

## 2 Materials and Methods

### 2.1 Sample Collection and DNA Extraction

**FIGURE 1:**
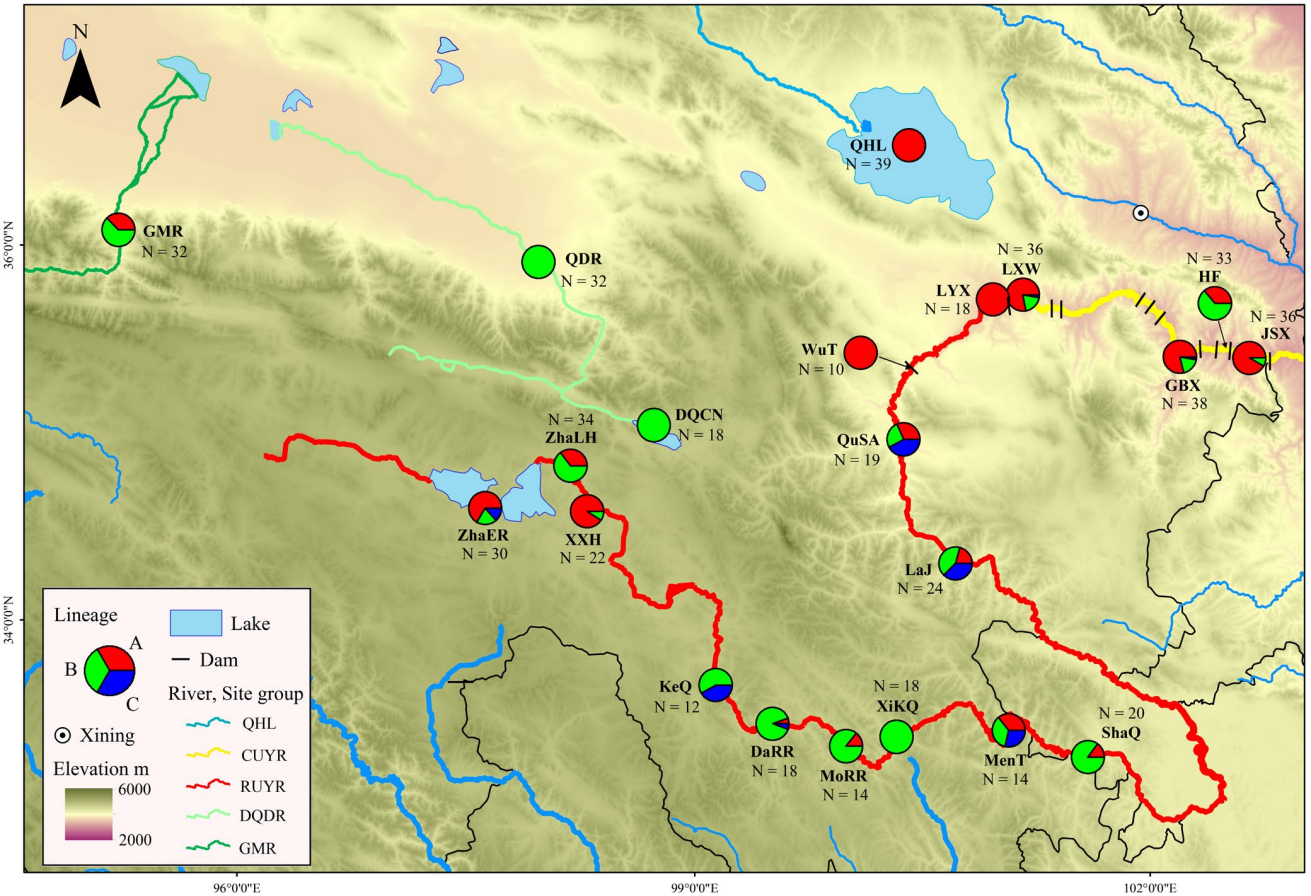
Distributions of sampling sites. The detailed information on the three lineages can be found in Figure 2 and the Result section, five site groups were represented by different line colors. CUYR, the canyon section of the upper Yellow River; DQDR, the Qaidam River; GMR, the Golmud River; *N*, number of samples; QHL, Qinghai Lake; RUYR, the river source section of the upper Yellow River.

A total of 517 samples of endemic Schizothoracinae fishes (i.e., *G. eckloni* , *G. przewalskii* , *S. pylzovi* , *S. kessleri* , *C. labiosa,* and *P. extremus* ) were collected from 21 sites of the upper Yellow River and its adjacent waters on the northeast QTP (Table 1). These sites were divided into five groups based on the watersheds and habitats: (i) Qinghai Lake (QHL), (ii) the canyon section of the upper Yellow River (CUYR, including five reservoirs: Jishixia, JSX; Huangfeng, HF; Gongboxia, GBX; Laxiwa, LXW; Longyangxia, LYX), (iii) the river source section of the upper Yellow River (RUYR: Wutuo, WuT; Qushian, QSA; Lajia, LaJ; Shaqu, ShaQ; Mentang, MenT; Xikequ, XiKQ; Mori, MoRR; DaRi, DaRR; Kequ, KeQ; Xingxinghai, XXH; Zhalinghu, ZhaLH; Zhaling_Eling riverway, ZhaER), (iv) Qaidam River (DQDR: Dongjicuona, DQCN; Qaidam River, QDR), and (v) Golmud River (GMR) (Figure 1). The number of samples of *G. eckloni* and *S. pylzovi* was 250 and 195, respectively. While *C. labiosa* and *P. extremus* had only eight and 25 individuals, respectively, and they were all from the sections above LaJ. In addition, ten samples of *Gymnodiptychus pachycheilus* , which belong to the specialized Schizothoracinae fishes, were collected from WuT and LaJ. A small piece of fin tissue was sampled from each fish individual and stored in absolute ethanol (Feng, Li, et al. 2023). Total genomic DNA of each sample was extracted from fin clips using a traditional phenol‐chloroform method (Taggart et al. 1992).

**TABLE 1:** Summary of population genetic diversity estimated using mitochondrial sequences.

Population	Group	*N*	COI sequence			D‐loop sequence			Concatenated sequence		
			*h*	*Hd*	*Pi*	*h*	*Hd*	*Pi*	*h*	*Hd*	*Pi*
Qinghai Lake (QHL)	QHL	39	6	0.617	0.00111	21	0.957	0.00718	22	0.958	0.00423
Jishixia (JSX)	CUYR	36	5	0.595	0.00735	11	0.832	0.00790	12	0.870	0.00764
Huangfeng (HF)	CUYR	33	8	0.780	0.01876	15	0.941	0.01463	15	0.941	0.01663
Gongboxia (GBX)	CUYR	38	8	0.802	0.01498	21	0.952	0.01378	22	0.953	0.01436
Laxiwa (LXW)	CUYR	36	8	0.759	0.01539	20	0.940	0.01514	20	0.940	0.01526
Longyangxia (LYX)	CUYR	18	5	0.484	0.00159	9	0.843	0.00819	10	0.882	0.00499
Wutuo (WuT)	RUYR	10	3	0.644	0.00190	5	0.756	0.00677	6	0.844	0.00441
Qushian (QuSA)	RUYR	19	7	0.854	0.02608	15	0.971	0.02642	15	0.971	0.02626
Lajia (LaJ)	RUYR	24	10	0.902	0.02477	19	0.982	0.02632	21	0.989	0.02557
Shaqu (ShaQ)	RUYR	20	6	0.632	0.01022	12	0.937	0.00979	13	0.942	0.01000
Mentang (MenT)	RUYR	14	6	0.857	0.02638	12	0.978	0.02713	12	0.978	0.02677
Xikequ (XiKQ)	RUYR	18	4	0.477	0.00103	10	0.876	0.00407	11	0.889	0.00260
Mori (MoRR)	RUYR	14	3	0.484	0.00977	7	0.824	0.00819	7	0.824	0.00895
DaRi (DaRR)	RUYR	18	7	0.739	0.00859	13	0.954	0.01056	15	0.974	0.00960
Kequ (KeQ)	RUYR	12	6	0.864	0.01904	11	0.985	0.02560	11	0.985	0.02242
Xingxinghai (XXH)	RUYR	22	6	0.684	0.00765	12	0.866	0.01063	13	0.896	0.00918
Zhalinghu (ZhaLH)	RUYR	34	6	0.777	0.01746	16	0.913	0.01520	18	0.938	0.01630
Zhaling_Eling Riverway (ZhaER)	RUYR	30	10	0.802	0.02031	17	0.931	0.02157	18	0.943	0.02096
Dongjicuona (DQCN)	DQDR	18	2	0.294	0.00088	5	0.817	0.00375	5	0.817	0.00236
Qaidam River (QDR)	DQDR	32	2	0.315	0.00094	8	0.841	0.00394	8	0.841	0.00249
Golmud River (GMR)	GMR	32	5	0.732	0.01846	6	0.764	0.01460	7	0.784	0.01647
Overall	—	517	38	0.833	0.02127	129	0.971	0.01882	146	0.976	0.02001

Abbreviations: CUYR, the canyon section of the upper Yellow River; DQDR, Qaidam River; GMR, Golmud River; *h*, number of haplotypes; *Hd*, haplotype diversity; *Pi*, nucleotide diversity; QHL, Qinghai Lake; RUYR, the river source section of the upper Yellow River.

### 2.2 PCR Amplification and Sequencing

The partial COI gene sequence was amplified using published primers (COI‐TriF: 5′‐TACCTGTGGCAATCACRCGCT‐3′, COI‐TriR: 5′‐ATTGTTGCRGAYGTAAARTATGC‐3′) (Feng et al. 2018). The partial D‐loop sequence was amplified using a newly designed primer pair (Gym_dloop‐F: 5′‐TTAACTCYCACCCCTGGCTC3′ and Gym_dloop‐R: 5′‐CRTCTTAGCATCWTCAGTGCTATG3′). The PCR was performed in a total volume of 25 μL, containing about 30 ng genomic DNA, 12.5 μL 2 × FTaq PCR MasterMix (ZomanBio, Beijing, China), 0.5 μL of each primer (10 μM), and the final volume was adjusted with sterile distilled water. The thermocycling parameters were as follows: an initial denaturation step at 94°C for 4 min, followed by 37 cycles of denaturation at 94°C for 30 s, annealing at 56°C for 35 s, extension at 72°C for 40 s, and a final extension at 72°C for 8 min. PCR products were then validated by agarose gel electrophoresis and were sequenced on an ABI Prism 3730 XL DNA analyzer (Applied Biosystems, Foster City, CA, USA).

### 2.3 Data Analysis

The sequences of COI and D‐loop were manually checked and edited, and aligned using the ClustalW algorithm (Thompson et al. 1994) implemented in MEGA 6 program (Tamura et al. 2013), and were trimmed to 666 and 710 bp, respectively. The aligned sequences of COI, D‐loop, and the concatenated sequences were analyzed separately. The genetic diversity indices including the number of polymorphic sites (*S*), number of haplotypes (*h*), haplotype diversity (*Hd*) and nucleotide diversity (*Pi*) were calculated for each population using DnaSP v5 (Librado and Rozas 2009). The phylogenetic trees based on haplotype sequences were constructed with the maximum parsimony (MP) algorithm by using MEGA 6 program, and the node support was assessed by the nonparametric bootstrap method with 1000 replicates. The haplotype networks were constructed using Popart v1.7 (Leigh and Bryant 2015) with the median‐joining method. The pairwise genetic differentiation (*F*_ST_) among populations was calculated using Arlequin v3.5.2.2 software (Excoffier and Lischer 2010), and the statistical significance was assessed through a bootstrapping analysis with 1000 replicates. Analysis of molecular variance (AMOVA) was performed to assess the distribution of genetic variation within and among populations and regions.

## 3 Results

### 3.1 Population Genetic Diversity

A total of 38, 129, and 146 haplotypes (GenBank accession no. PQ620059–PQ620097 and PQ621487–PQ621619) were detected from COI, D‐loop, and their concatenated sequences, respectively (Table 1). In addition, one, four, and four haplotypes were detected in samples of *G. pachycheilus* . Since most species widely shared haplotypes (except for *G. pachycheilus* , Figure S1), and previous studies have demonstrated very close genetic relationships among the genus *Gymnocypris*, *Schizopygopsis*, and *Chuanchia* (Qi et al. 2006; Tang et al. 2019), all species were treated as a single species for data analysis except for *G. pachycheilus,* which was designated as the outgroup. Based on the concatenated sequences, the haplotype diversity (*Hd*) of all populations ranged from 0.784 to 0.989 (mean: 0.912), and the nucleotide diversity (*Pi*) ranged from 0.00236 to 0.02677 (mean: 0.01274). Populations from the upper Yellow River (mean *Hd*: 0.927, mean *Pi*: 0.01423) and Qinghai Lake (*Hd*: 0.958, *Pi*: 0.00423) showed a higher genetic diversity than populations from the Qaidam River (mean *Hd*: 0.829, mean *Pi*: 0.00243). Although the Golmud River population had the lowest haplotype diversity (*Hd*: 0.784), its nucleotide diversity was higher (*Pi*: 0.01647) than that of most populations. The average genetic diversity of populations from the canyon section of the upper Yellow River (mean *Hd*: 0.917 and *Pi*: 0.01178) was slightly lower than that of populations from the river source section of the upper Yellow River (mean *Hd*: 0.931 and *Pi*: 0.01525), but there was no statistically significant difference between them (two‐sample *t*‐test, *p* > 0.05). Furthermore, in the upper Yellow River, the three dam‐affected populations (JSX, LYX, and WuT), which received few stock enhancements, exhibited lower genetic diversity (*Hd*: 0.844–0.882) than populations from the undammed upstream sections (mean *Hd*: 0.939), while the other three dam‐affected populations (HF, GBX, and LXW), which received frequent stocking events showed a relatively high genetic diversity (*Hd*: 0.940–0.953). Similar results were also obtained based on COI and D‐loop sequences.

### 3.2 Phylogeographic Relationships Among Haplotypes

**FIGURE 2:**
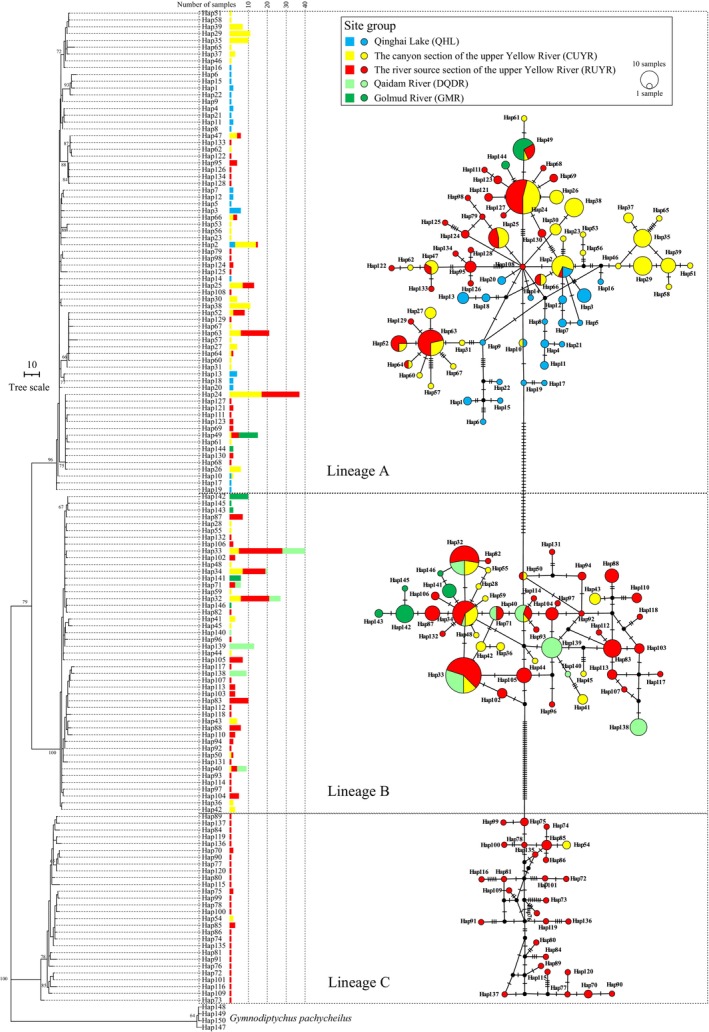
The maximum parsimony phylogenetic tree (left) and median‐joining network (right) constructed based on haplotypes of concatenated sequences. Numbers at the nodes indicate bootstrap values based on 1000 replications.

The maximum parsimony phylogenetic tree constructed based on concatenated sequences showed that all haplotypes were divided into three distinct lineages, i.e., lineage A, B, and C, containing 71, 47, and 28 haplotypes, respectively (Figure 2). All samples from Qinghai Lake belonged to lineage A, while all samples from Qaidam River belonged to lineage B. Samples from Golmud River were assigned to both lineage A and B, and samples from the upper Yellow River were assigned to all three lineages. The lineage C was only represented by samples from the upper Yellow River, with the majority (93.5%) of samples from the river source section and only two samples from the canyon section. The median‐joining haplotype network showed that all haplotypes were clustered into three groups, corresponding to the three lineages of the phylogenetic tree. The phylogenetic trees and median‐joining networks constructed based on haplotypes of both COI and D‐loop sequences also showed the presence of three distinct lineages, which were consistent with results from the concatenated sequences (Figures S2 and S3).

### 3.3 Genetic Differentiation and Population Genetic Structure

Based on the concatenated sequences, the pairwise genetic distances among geographical populations ranged from 0.0024 to 0.0378 (mean: 0.0222), with the maximum value between LYX and KeQ and the minimum value between DGCN and QDR (Table 2). The genetic differentiation coefficients (*F*_ST_) among geographical populations ranged from −0.0420 to 0.9048 (mean: 0.2989), with the highest value between WuT and QDR and the lowest value between ShaQ and MoRR. The *F*_ST_ between populations within groups (mean: 0.2510) was significantly lower than those between groups (mean: 0.3737), while there was no significant difference in genetic distances (mean: 0.0223 vs. 0.0221). For the group analysis, the highest *F*_ST_ was observed between Qinghai Lake and Qaidam River (0.8976), and the lowest *F*_ST_ was detected between Golmud River and the river source section of the upper Yellow River (0.0329). Additionally, relatively low levels of genetic differentiation were also detected between Qinghai Lake and the canyon section of the upper Yellow River (*F*_ST_: 0.1322), as well as between the river source section and the canyon section of the upper Yellow River (*F*_ST_: 0.1585), while relatively high levels of genetic differentiation were detected between Qinghai Lake and Golmud River (0.5526) and between Qaidam River and the canyon section of the upper Yellow River (0.5625) (Table 3).

**TABLE 2:** Pairwise genetic distance (above the diagonal) and genetic differentiation (*F*_ST_: below the diagonal) among populations based on the concatenated sequences of COI and D‐loop.

Population	QHL	JSX	HF	GBX	LXW	LYX	WuT	QSA	LaJ	ShaQ	MenT	XiKQ	MoRR	DaRR	KeQ	XXH	ZhaLH	ZhaER	DGCN	QDR	GMR
QHL		0.0067	0.0229	0.0114	0.0121	0.0055	0.0058	0.0285	0.0310	0.0281	0.0263	0.0321	0.0284	0.0316	0.0374	0.0081	0.0230	0.0167	0.0326	0.0324	0.0235
JSX	0.0912		0.0216	0.0121	0.0129	0.0072	0.0068	0.0277	0.0299	0.0258	0.0255	0.0291	0.0261	0.0289	0.0354	0.0088	0.0214	0.0167	0.0296	0.0294	0.0217
HF	0.5320	0.4111		0.0215	0.0217	0.0232	0.0228	0.0276	0.0260	0.0149	0.0250	0.0136	0.0146	0.0158	0.0257	0.0224	0.0171	0.0241	0.0136	0.0136	0.0177
GBX	0.1518	0.0515	0.2390		0.0153	0.0118	0.0117	0.0283	0.0297	0.0247	0.0262	0.0270	0.0248	0.0272	0.0338	0.0130	0.0217	0.0194	0.0274	0.0272	0.0222
LXW	0.1654	0.0734	0.2244	−0.0210		0.0123	0.0124	0.0288	0.0301	0.0248	0.0265	0.0270	0.0249	0.0274	0.0341	0.0139	0.0220	0.0200	0.0274	0.0272	0.0227
LYX	0.1534	0.0838	0.4654	0.1147	0.1112		0.0048	0.0287	0.0316	0.0283	0.0263	0.0323	0.0288	0.0319	0.0378	0.0082	0.0231	0.0164	0.0329	0.0327	0.0237
WuT	0.2454	0.0646	0.4326	0.1043	0.1124	0.0007		0.0282	0.0312	0.0278	0.0259	0.0318	0.0283	0.0314	0.0373	0.0075	0.0224	0.0158	0.0323	0.0321	0.0228
QSA	0.5057	0.3939	0.1849	0.2533	0.2443	0.4091	0.3537		0.0270	0.0267	0.0274	0.0264	0.0266	0.0269	0.0272	0.0280	0.0272	0.0282	0.0269	0.0268	0.0276
LaJ	0.5365	0.4353	0.1397	0.2933	0.2839	0.4542	0.4056	−0.0243		0.0238	0.0274	0.0226	0.0237	0.0236	0.0252	0.0302	0.0257	0.0294	0.0229	0.0228	0.0262
ShaQ	0.7641	0.6517	0.0533	0.4647	0.4467	0.7191	0.7005	0.2855	0.1986		0.0238	0.0069	0.0095	0.0101	0.0214	0.0265	0.0147	0.0259	0.0070	0.0070	0.0155
MenT	0.4929	0.3568	0.0933	0.1930	0.1811	0.3814	0.3210	−0.0340	−0.0209	0.2073		0.0234	0.0238	0.0244	0.0274	0.0260	0.0245	0.0267	0.0238	0.0237	0.0253
XiKQ	0.8814	0.7915	0.2088	0.6146	0.5981	0.8790	0.8947	0.4205	0.3098	0.0498	0.3749		0.0063	0.0065	0.0184	0.0299	0.0132	0.0277	0.0026	0.0025	0.0142
MoRR	0.7921	0.6746	0.0586	0.4754	0.4558	0.7541	0.7409	0.2805	0.1950	−0.0420	0.2087	0.0546		0.0095	0.0210	0.0270	0.0145	0.0263	0.0063	0.0063	0.0152
DaRR	0.7989	0.6962	0.1103	0.5163	0.4995	0.7608	0.7449	0.2924	0.1938	−0.0201	0.2286	0.0163	−0.0223		0.0202	0.0296	0.0156	0.0279	0.0064	0.0064	0.0163
KeQ	0.7269	0.6220	0.2014	0.4499	0.4344	0.6484	0.5966	0.0428	−0.0162	0.2167	0.0417	0.3318	0.2130	0.1774		0.0356	0.0252	0.0324	0.0186	0.0186	0.0261
XXH	0.1670	0.0128	0.3756	0.0456	0.0733	0.1094	0.0431	0.3321	0.3778	0.6207	0.2932	0.7845	0.6462	0.6664	0.5620		0.0219	0.0170	0.0304	0.0302	0.0221
ZhaLH	0.5427	0.4155	−0.0114	0.2574	0.2465	0.4737	0.4359	0.1829	0.1374	0.0486	0.0857	0.2044	0.0642	0.1085	0.1973	0.3732		0.0236	0.0135	0.0134	0.0174
ZhaER	0.2205	0.1020	0.1781	0.0365	0.0420	0.1357	0.0887	0.1079	0.1555	0.3539	0.0541	0.5054	0.3615	0.3972	0.2880	0.0566	0.1705		0.0281	0.0280	0.0242
DGCN	0.8853	0.7974	0.2193	0.6223	0.6058	0.8845	0.9012	0.4334	0.3229	0.0711	0.3899	0.0221	0.0730	0.0202	0.3479	0.7911	0.2223	0.5139		0.0024	0.0143
QDR	0.8903	0.8160	0.2589	0.6605	0.6467	0.8921	0.9048	0.5026	0.3831	0.0888	0.4664	−0.0040	0.0941	0.0438	0.4223	0.8165	0.2597	0.5674	−0.0277		0.0142
GMR	0.5526	0.4215	0.0162	0.2686	0.2609	0.4849	0.4401	0.1886	0.1492	0.0919	0.1116	0.2473	0.0968	0.1405	0.2194	0.3739	0.0066	0.1818	0.2610	0.3008	

**TABLE 3:** Pairwise genetic distance (above the diagonal) and genetic differentiation (*F*_ST_: below the diagonal) among groups based on the concatenated sequences of COI and D‐loop.

Group	QHL	CUYR	RUYR	DQDR	GMR
QHL		0.0122	0.0244	0.0325	0.0235
CUYR	0.1322		0.0231	0.0256	0.0215
RUYR	0.3427	0.1585		0.0182	0.0209
DQDR	0.8976	0.5625	0.2226		0.0143
GMR	0.5526	0.2324	0.0329	0.3501	

Abbreviations: CUYR, the canyon section of the upper Yellow River; DQDR, Qaidam River; GMR, Golmud River; QHL, Qinghai Lake; RUYR, the river source section of the upper Yellow River.

The AMOVA analysis based on the concatenated sequences showed that the genetic variation within populations accounted for 58.58% of the total variation, which was higher than the proportions of genetic variation among groups (19.25%) and among populations within groups (22.16%), indicating that genetic variation was mainly from within populations (Table 4). When analyzing only samples from the river source section and the canyon section of the upper Yellow River, genetic variation within populations, among groups, and among populations within groups accounted for 64.80%, 12.55% and 22.65%, respectively. Similar results of genetic differentiation and population structure were obtained based on the analysis of COI and D‐loop sequences (Tables S1–S3).

**TABLE 4:** Analysis of molecular variances (AMOVA) based on the concatenated sequences of COI and D‐loop.

Source of variation	df	Sum of squares	Variance components	Percentage of variation	Fixation index
Among groups (QHL, CUYR, RUYR, DQDR, GMR)	4	1475.781	2.90973 Va	19.25	*F*_ct_ = 0.19252**
Among populations within groups	16	1358.449	3.34985 Vb	22.16	*F*_sc_ = 0.27448**
Within populations	496	4391.776	8.85439 Vc	58.58	*F*_st_ = 0.41416**
Total	516	7226.006	15.11397		

Abbreviations: CUYR, the canyon section of the upper Yellow River; DQDR, Qaidam River; GMR, Golmud River; QHL, Qinghai Lake; RUYR, the river source section of the upper Yellow River.

** Significant level at *p* < 0.01.

## 4 Discussion

Our study investigated genetic diversity, phylogenetic relationships, and population genetic structure of the endemic Schizothoracinae fishes in the upper Yellow River and its adjacent waters using mitochondrial COI and D‐loop sequences. The upper Yellow River and Qinghai Lake populations exhibited higher genetic diversity than the isolated populations from the Qaidam River and Golmud River, with all three lineages present only in the upper Yellow River versus one or two lineages in others. Our results highlight that habitat isolation and connectivity caused by paleogeological events play key roles in driving population genetic structure, and human activities can have profound impacts on population genetic diversity.

Geological studies have demonstrated connections and isolations between various basins on the northeastern QTP during the Late Pliocene and early Pleistocene, while the formation and geomorphological evolution of the upper Yellow River is relatively recent compared to other rivers and lakes in the region (Li et al. 2000; Zhang et al. 2003; Chang et al. 2009; Qi 2016). From about 1.1 million to 0.03 million years ago, the upper Yellow River experienced successive phases of headward erosion, which ultimately extended its drainage to the current source region. The connectivity and isolation between river and basin water systems directly influence the isolation and dispersal of fish species, thereby shaping their population genetic structure. In this study, populations of the endemic Schizothoracinae fishes from Qinghai Lake and the Qaidam River (endorheic systems) both exhibited only one single and distinct lineage each. However, another isolated population from the Golmud River and populations from the upper Yellow River possessed a mixed lineage composition. Based on such genetic patterns, the following inferences may be drawn: (i) the ancient populations from Qinghai Lake and the canyon section of the upper Yellow River belonged to lineage A, while the ancient populations from Golmud River, Qaidam River and the river source section of the upper Yellow River belonged to lineage B, and lineage A and B were mixed after the connection between the river source section and canyon section; (ii) assuming the separation times of Qinghai Lake, Qaidam River and Golmud River from the upper Yellow River are T1, T2, and T3, respectively, and the connection time between the river source section and the canyon section is T4, T1 and T2 may be earliest, followed by T4 and then T3; (iii) there may be other more recent events leading to lineage C entering the upper Yellow River from other basins which were not included in this study. Further studies on population genomics are needed to verify these inferences and estimate the timing of these events.

Habitat fragmentation driven by dam construction, resulting in loss of genetic diversity and population genetic differentiation of fishes, has been reported in some studies (Horreo et al. 2011; Gouskov et al. 2016; Klütsch et al. 2019; He et al. 2024). Similarly, in the upper Yellow River of this study, the dam‐affected populations, which received very few stock enhancements, showed lower genetic diversity than populations from the undammed upstream reaches, indicating the loss of genetic diversity driven by dam construction. However, the other three dam‐affected populations, which received frequent stock enhancement exhibited a relatively high genetic diversity, demonstrating that artificial stock enhancement mitigates the negative fragmentation effects of dam construction. Such effects of stocking and hydropower on population genetic diversity were also found in the study of brown trout in the Pasvik River, which showed low genetic diversity in nonstocked parts and relatively high across stocked sections (Klütsch et al. 2019). These findings highlighted the critical role of stocking activities in maintaining the genetic diversity of dam‐affected populations and suggested that populations with low genetic diversity require more stocking efforts.

The level of genetic differentiation in the dam‐affected sections was comparable to that in the undammed sections (mean *F*_ST_ between adjacent populations: 0.1482 vs. 0.1380), indicating that the hydropower dams did not seem to cause genetic differentiation in the fish populations. One explanation for this is that stocking promoted genetic homogenization, despite the considerable variation in its scale across different river sections. Another potential explanation is that the dam's impact on population genetic differentiation may exhibit a time lag, potentially becoming detectable after dozens of generations (Ruzich et al. 2019). The genetic homogenization potentially caused by stocking may be further elucidated by comparing our findings with previous studies. Based on mitochondrial sequences of samples collected before 2009, previous studies revealed high levels of genetic differentiation and almost no shared haplotypes between populations from the canyon section and headwaters of the upper Yellow River (Zhao et al. 2005, 2009). However, in this study, they exhibited lower genetic differentiation and numerous shared haplotypes, which were likely caused by stocking practices using broodstock from headwaters, thereby promoting genetic homogenization along the river. Notably, all haplotypes from previous studies belonged to a single lineage in this study (Figure S4), suggesting that the current data provided limited evidence for the easily detectable influence of stocking on genetic homogenization.

The Qinghai Lake and Golmud River populations represent two other cases severely impacted by human activities (overfishing and dam construction). The resource of the former has received extensive stock supplementation, whereas the latter has not. As anticipated, the Qinghai Lake population exhibited high haplotype diversity (*Hd*_COI+D‐loop_: 0.958), in stark contrast to the low diversity observed in the Golmud River population (*Hd*_COI+D‐loop_: 0.784). However, on the other hand, the Qinghai Lake population had a relatively low nucleotide diversity (*Pi*_COI+D‐loop_: 0.00423), and the Golmud River populations possessed a relatively high nucleotide diversity (*Pi*_COI+D‐loop_: 0.01647), which may be related to the historical events they have experienced. Historical events such as tectonic uplift and river headward erosion (Zhang et al. 2003) have resulted in only one lineage in the Qinghai Lake population and two lineages in the Golmud River population, and the mixture of different lineages generally has higher nucleotide diversity (Soares et al. 2018; Feng, Liu, et al. 2023). The low genetic diversity observed in the Qaidam River population is likely attributable to its extreme environmental conditions. Most water bodies in the Qaidam Basin exhibit high salinity levels (Zhang et al. 2019), which have compressed the suitable habitat for the Schizothoracinae fishes to sediment‐laden rivers and few small lakes.

One limitation of this study is the use of mitochondrial DNA markers that are maternally inherited and represent only a small fraction of the total genomic variation. They lack the power to analyze certain deeper aspects, such as signals of hybridization between distinct lineages following historical connectivity of the upper Yellow River, fluctuations in recent effective population size across different water bodies, the timing of genetic divergence, and the loss of low‐frequency alleles in populations after stocking. Future studies should utilize population genomic analyses based on genome‐wide SNP markers to validate and address these questions. On the other hand, mitochondrial DNA markers are also widely employed in population genetic studies and are cost‐effective for DNA sequencing, making them suitable for analysis of large numbers of samples. The mitochondrial and nuclear discordance in biogeography analyses (Toews and Brelsford 2012) underscores that mitochondrial markers constitute an indispensable component of population genetic analysis. For instance, in this study, the detection of three distinct lineages within the contiguous waters of the upper Yellow River might have been missed by nuclear genetic markers alone.

## 5 Conclusion

The present study investigated genetic diversity and population genetic structure of the endemic Schizothoracinae fishes in the upper Yellow River and its adjacent waters by using mitochondrial genes. The results demonstrated that populations from the upper Yellow River and Qinghai Lake had a higher genetic diversity than the isolated populations from the Qaidam River and Golmud River. Genetic diversity in the upper Yellow River differed between dam‐affected populations with few and frequent stocking events. Furthermore, populations from the upper Yellow River possessed all three lineages, while populations from other waters had only one or two lineages. Our results indicated that habitat isolation and connectivity caused by paleogeological events primarily drove the formation of the population genetic structure, and human activities such as dam construction and stocking activities have had a profound impact on population genetic diversity.

## Author Contributions

**Xiu Feng:** data curation (equal), formal analysis (equal), investigation (equal), methodology (equal), software (equal), validation (equal), visualization (lead), writing – original draft (equal), writing – review and editing (equal). **Xiaoling Wang:** data curation (equal), formal analysis (equal), investigation (equal), methodology (equal), software (equal), validation (equal), writing – original draft (equal). **Ren Zhu:** data curation (equal), formal analysis (equal), investigation (equal), project administration (equal), resources (equal), validation (equal), writing – review and editing (equal). **Yintao Jia:** data curation (equal), formal analysis (equal), investigation (equal), resources (equal), validation (equal), writing – review and editing (equal). **Xiaoyun Sui:** data curation (equal), formal analysis (equal), investigation (equal), project administration (equal), validation (equal), writing – review and editing (equal). **Yu Zhuo:** data curation (equal), project administration (equal), validation (equal), writing – review and editing (equal). **Junle Li:** data curation (equal), investigation (equal), resources (equal). **Yifeng Chen:** conceptualization (lead), funding acquisition (lead), methodology (equal), supervision (equal), validation (equal), writing – review and editing (equal).

## Conflicts of Interest

The authors declare no conflicts of interest.

## Supporting information


**Figure S1:** The median‐joining networks constructed based on mitochondrial COI (a), D‐loop (b) and concatenated (c) haplotypes for all species including the outgroup, *Gymnodiptychus pachycheilus* . Panels a, b, and c shared a common legend.

**Figure S2:** The maximum parsimony phylogenetic tree (left) and median‐joining network (right) constructed based on mitochondrial COI haplotypes. Numbers at the nodes indicate bootstrap values based on 1000 replications.

**Figure S3:** The maximum parsimony phylogenetic tree (left) and median‐joining network (right) constructed based on mitochondrial D‐loop haplotypes. Numbers at the nodes indicate bootstrap values based on 1000 replications.

**Figure S4:** The median‐joining network constructed based on mitochondrial D‐loop haplotypes for samples in this study and a previously published study (Zhao et al. 2009, Mol. Ecol. 18, 3616–3628).

**Table S1:** Pairwise genetic distance (above the diagonal) and genetic differentiation (*F*_ST_: below the diagonal) among populations based on mitochondrial COI and D‐loop sequences.
**Table S2:** Pairwise genetic distance (above the diagonal) and genetic differentiation (*F*_ST_: below the diagonal) among groups based on mitochondrial COI and D‐loop sequences.
**Table S3:** Analysis of molecular variances (AMOVA) based on mitochondrial COI and D‐loop sequences.

## Data Availability

The sequences of mitochondrial COI and D‐loop haplotypes have been deposited in GenBank (https://www.ncbi.nlm.nih.gov/) with accession no. PQ620059–PQ620097 and PQ621487–PQ621619, respectively.
