# Supplementary figures and images for: Genetic Diversity and Population Genetic Structure of Endemic Schizothoracinae Fishes in the Upper Yellow River and Its Adjacent Waters
Source: Ecol Evol. 2025 Dec 18;15(12):e72813. doi: 10.1002/ece3.72813 (PMC12714410; doi:10.1002/ece3.72813)

(a)

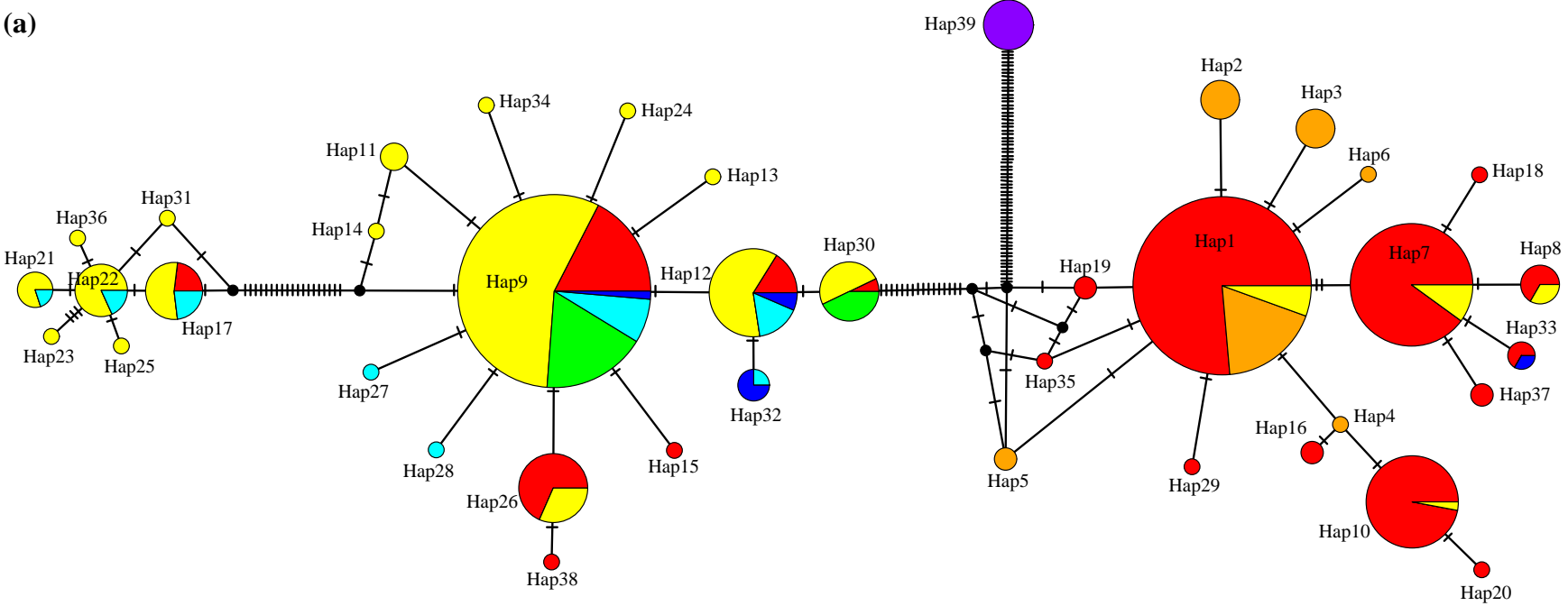

(b)

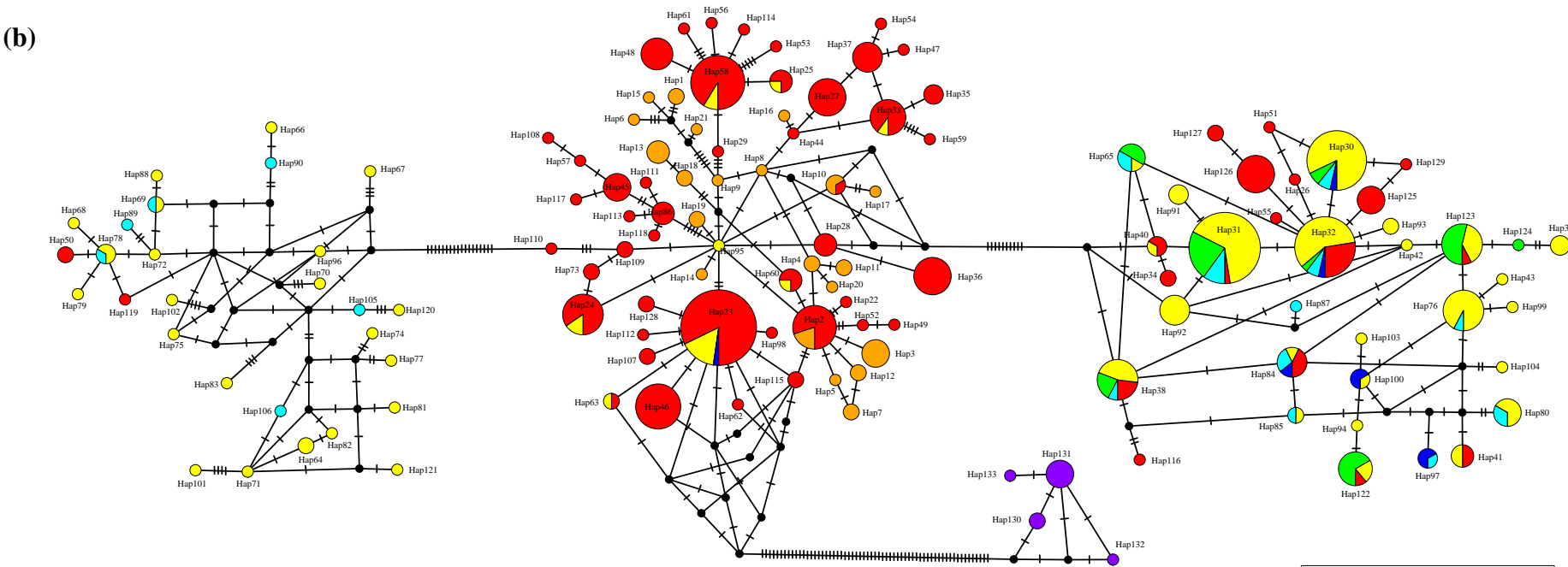

(c)

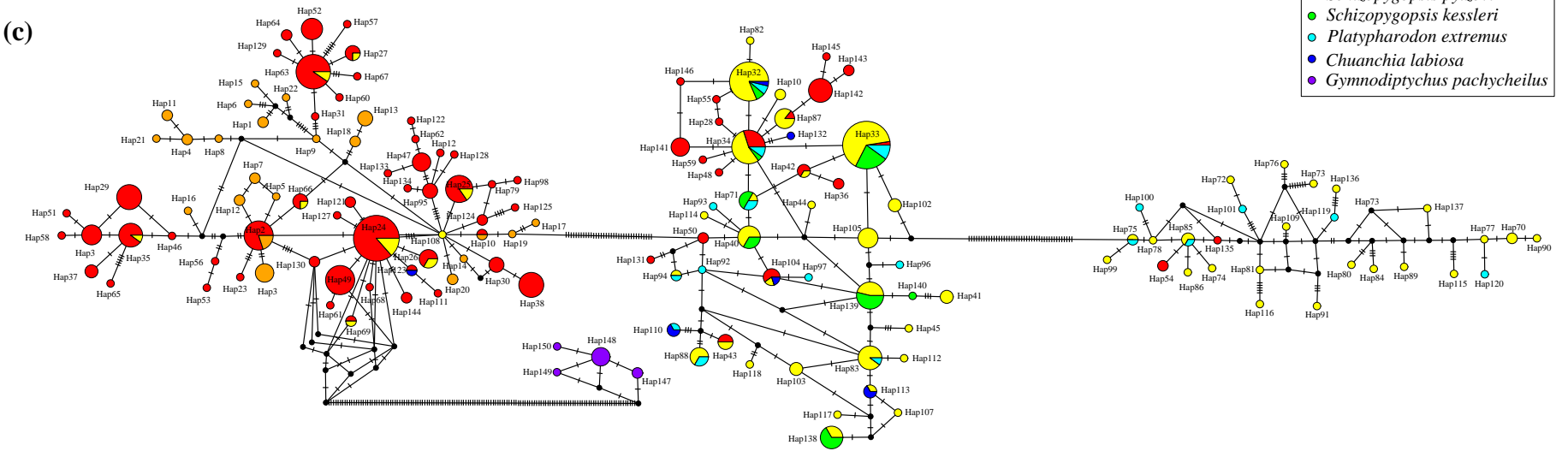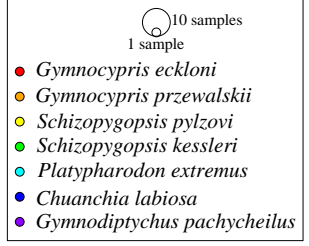

Supplement: Supplementary file 1 — Figure S1: The median‐joining networks constructed based on mitochondrial COI (a), D‐loop (b) and concatenated (c) haplotypes for all species including the outgroup, Gymnodiptychus pachycheilus . Panels a, b, and c shared a common legend. [file ECE3-15-e72813-s001.pdf]

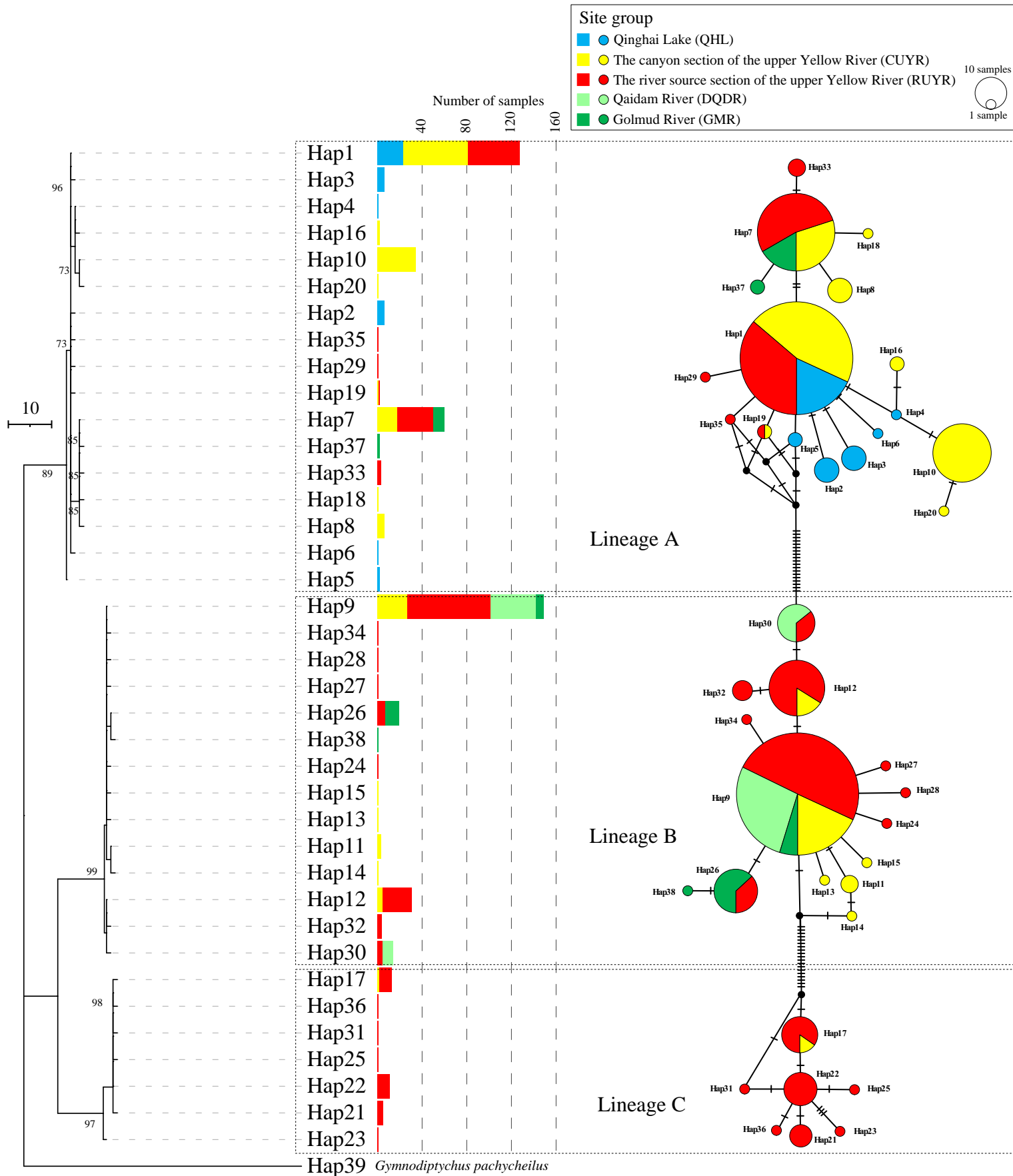

Supplement: Supplementary file 2 — Figure S2: The maximum parsimony phylogenetic tree (left) and median‐joining network (right) constructed based on mitochondrial COI haplotypes. Numbers at the nodes indicate bootstrap values based on 1000 replications. [file ECE3-15-e72813-s005.pdf]

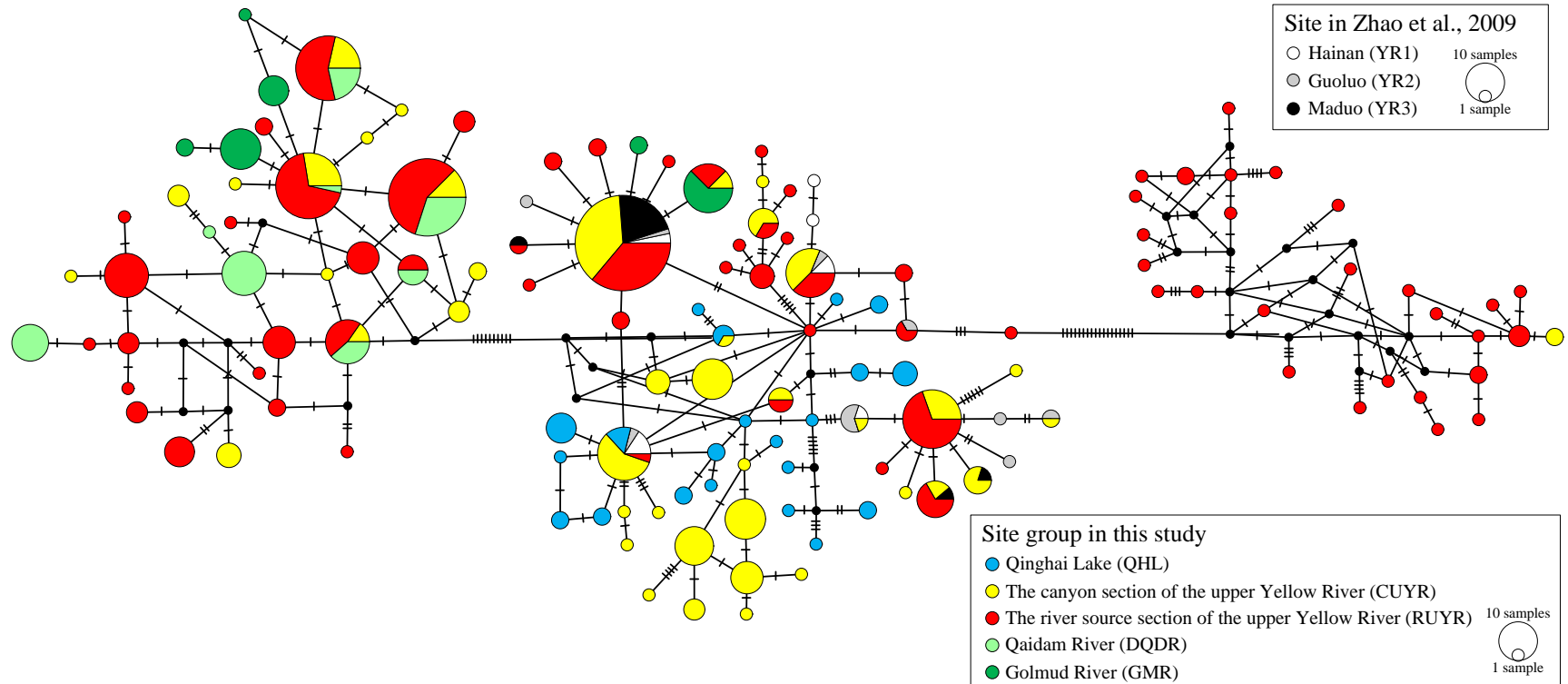

Supplement: Supplementary file 4 — Figure S4: The median‐joining network constructed based on mitochondrial D‐loop haplotypes for samples in this study and a previously published study (Zhao et al. 2009, Mol. Ecol. 18, 3616–3628). [file ECE3-15-e72813-s002.pdf]
