# Supplementary material for: Genetic Diversity and Population Genetic Structure of Endemic Schizothoracinae Fishes in the Upper Yellow River and Its Adjacent Waters
Source: Ecol Evol. 2025 Dec 18;15(12):e72813. doi: 10.1002/ece3.72813 (PMC12714410; doi:10.1002/ece3.72813)
Supplement: Supplementary file 3 — Figure S3: The maximum parsimony phylogenetic tree (left) and median‐joining network (right) constructed based on mitochondrial D‐loop haplotypes. Numbers at the nodes indicate bootstrap values based on 1000 replications. [file ECE3-15-e72813-s004.pdf]

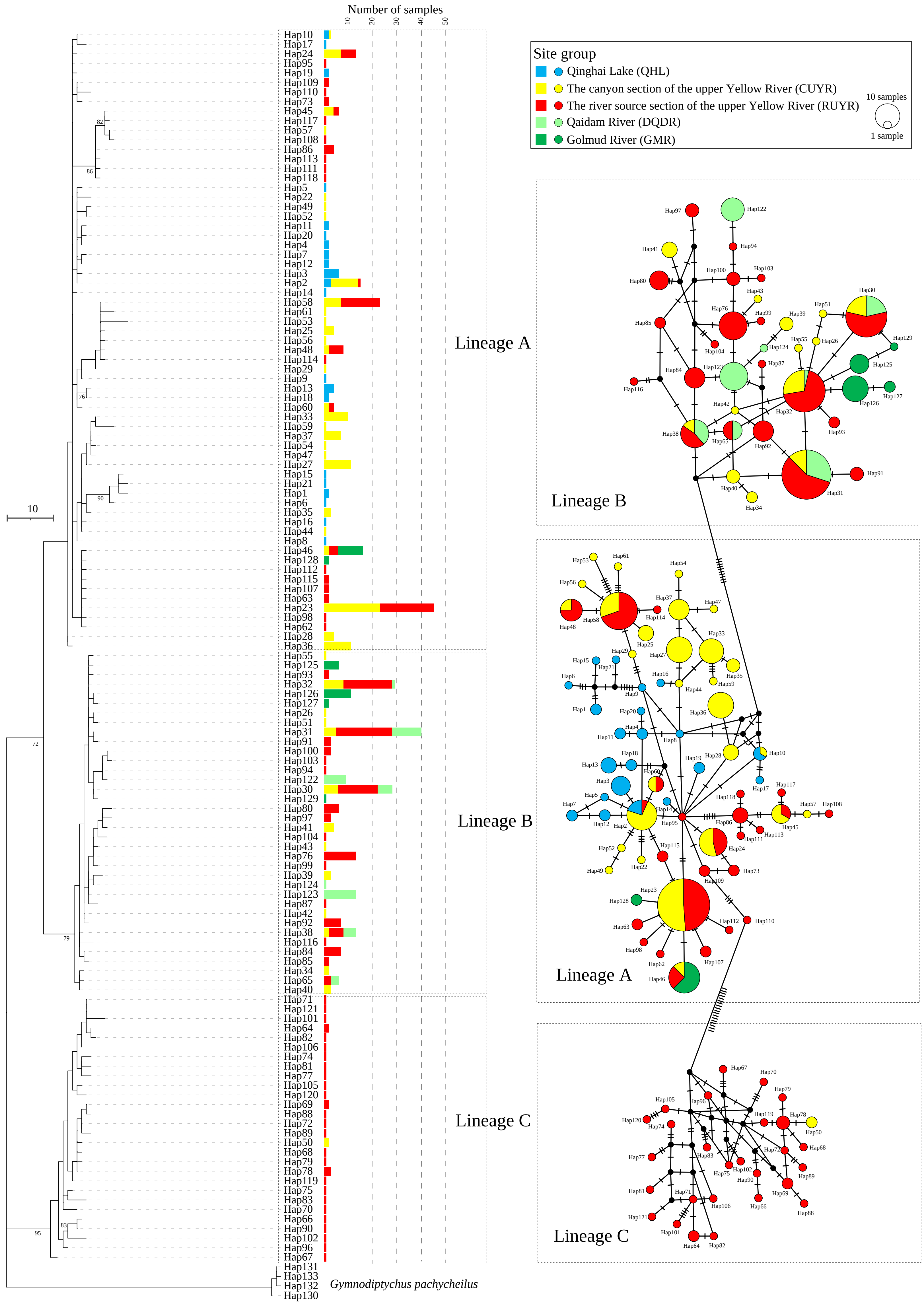

Number of samples
10
20
30
40
50
Site group
Qinghai Lake (QHL)
The canyon section of the upper Yellow River (CUYR)
The river source section of the upper Yellow River (RUYR)
Qaidam River (DQDR)
Golmud River (GMR)
10 samples
1 sample
Lineage A
Lineage B
Lineage C
Gymnodiptychus pachycheilus
10
